# Review of Recent Laboratory and Experimental Data on Cardiotoxicity of Statins

**DOI:** 10.3390/jcdd9110403

**Published:** 2022-11-19

**Authors:** Aleksey M. Chaulin

**Affiliations:** 1 Department of Histology and Embryology, Samara State Medical University, 443099 Samara, Russia; alekseymichailovich22976@gmail.com; Tel.: +7-(927)-770-25-87; 2 Department of Cardiology and Cardiovascular Surgery, Samara State Medical University, 443099 Samara, Russia; 3 Research Institute of Cardiology, Samara State Medical University, 443099 Samara, Russia

**Keywords:** statins, laboratory diagnostics, cardiac troponins, cardiotoxicity, mechanisms of cardiotoxicity

## Abstract

Due to the fact that statins are among the most high-demand therapeutic agents used for the treatment and prevention of the most common cardiovascular diseases, a significant amount of research is focused on these drugs. As a result, the study and discovery of new effects in statin drugs continues. Research methods are constantly being improved in terms of their sensitivity and specificity, which leads to a change in ideas. In addition to the main lipid-lowering effect, statins have a number of additional effects, which can be conditionally divided into positive (pleiotropic) and negative (side effects). Moreover, information about many of the pleiotropic effects of statins is controversial and may subsequently change as new data become available. To a large extent, this is due to the introduction of new and the improvement of old methods of study: clinical, laboratory and morphological ones. Recent studies report the possibility of statins having potential cardiotoxic properties, which is expressed by an increase in the concentration of highly sensitive cardiac troponins, as well as various adverse changes in cardiac myocytes at the ultrastructural and molecular levels. This paper discusses possible mechanisms of statin cardiotoxicity. This narrative review is based on an analysis of publications in the Medline, PubMed, PubMed Central and Embase databases. The terms “statins”, “troponin”, “troponin I”, “troponin T” in combination with “cardiotoxicity”, “false positive”, “mechanisms of increase”, “pathophysiological mechanisms”, “oxidative stress” and “cardiomyocyte apoptosis” were used to search publications.

## 1. Introduction

Since Russian scientists A.I. Ignatowski and N.N. Anichkov emphasized the importance of cholesterol in the pathophysiology of atherosclerosis and cardiovascular diseases, the efforts of most researchers have been focused on the development of drugs that lower the level of cholesterol in blood serum [1,2,3]. Inspired by the discovery of penicillin by A. Fleming, many researchers and pharmacological companies began to actively study substances synthesized by fungi and various microorganisms [4]. As a result of such studies, in 1971 the Japanese scientist A. Endo discovered that the waste products of fungi have a lipid-lowering effect. The first statin drug ML 236B, isolated from the culture medium of *Penicillium citrinium* and named compactin, competitively inhibited the key enzyme of cholesterol biosynthesis—3-hydroxy-3-methylglutaryl-coenzyme A (HMG-CoA)—reductase [4,5]. Subsequent clinical trials have demonstrated high efficiency of statins, and to date they remain the main lipid-lowering drugs for patients in terms of treating and preventing atherosclerosis and cardiovascular disease (CVD) [6,7,8].

In terms of the effectiveness and safety of lipid-lowering, statins are second only to inhibitors of proprotein convertase subtilisin/kexin type 9 (PCSK-9) [9,10,11]. This enzyme, which belongs to the serine class, is an important participant in the metabolism of low-density lipoproteins by regulating the density of low-density-lipoprotein receptors in the hepatocyte cell membrane [12,13]. However, despite the fact that some drugs that inhibit PCSK-9 (monoclonal PCSK-9 antibodies—alirocumab and evolocumab) have been approved by leading foreign authorities (US FDA and Europe EMEA) for practical use in the near future, due to economic reasons, they are unlikely to have the same high prevalence as statins. Thus, this paper reports that the annual cost of treatment is USD 14,000–15,000, which is unprofitable. According to researchers, to achieve economic feasibility, the cost of drugs that inhibit PCSK-9 should be reduced by at least 70% [14]. Since such a reduction in the cost of treatment is not expected in the near future, statins will continue to be the main lipid-lowering drugs for the majority of the population. 

Recent studies have reported that some drugs, including statins, can cause a CT increase in serum, potentially indicative of statin cardiotoxicity. However, the specific mechanisms underlying the statin-induced increase in CTs and statin cardiotoxicity are unknown. The purpose of this article is to discuss the possible mechanisms of the statin-induced increase in CTs and statin cardiotoxicity.

### Materials and Methods

This narrative review is based on an analysis of publications in the Medline, PubMed, PubMed Central and Embase databases. The terms “statins”, “troponin”, “troponin I”, “troponin T” in combination with “cardiotoxicity”, “false positive”, “mechanisms of increase”, “pathophysiological mechanisms”, “oxidative stress” and “cardiomyocyte apoptosis” were used to search publications. The main criteria for inclusion and exclusion of articles are presented in Table 1.

## 2. Effects of Statins

Statins, in addition to their main lipid-lowering effect, have many additional (non-lipid) both favorable (pleiotropic) and unfavorable (toxic) effects. The favorable (pleiotropic) effects of statins on the cardiovascular system include: antifibrotic and antihypertrophic effects [18,19], anti-inflammatory effects [20], antiapoptotic effects [21,22], and antioxidant properties [23,24]. Due to these beneficial effects, statin drugs can be used as cardioprotectors during and after cancer chemotherapy [25,26]. However, it is worth noting the presence of a lot of conflicting data on the favorable (pleiotropic) properties of statins. For example, the antioxidant and anti-inflammatory properties of statins have not been found in several studies [27,28]. On the contrary, some researchers have reported that statins have pro-oxidant rather than antioxidant effects [29,30,31]. Thus, further studies are needed to finally confirm or refute the mechanisms of the pleiotropic (antioxidant) effect of statins.

**Figure 1:**
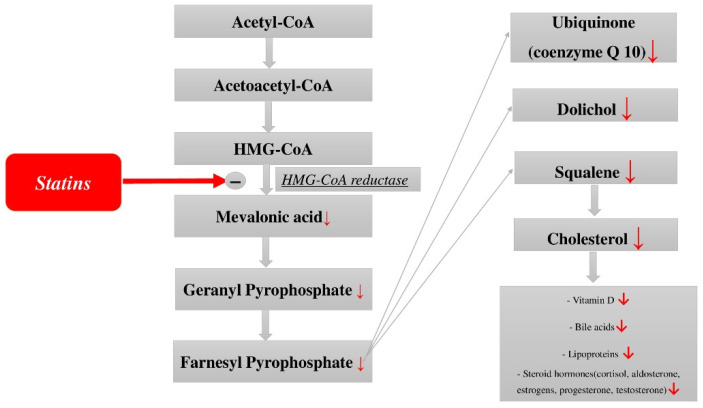
Scheme of cholesterol biosynthesis and its metabolites.

A number of toxic effects of statins are also known, which force doctors to cancel or reduce the dose of the drug and can cause dangerous complications. The latter include hepatotoxicity, diabetogenic effect, myotoxicity, and a number of others [32,33,34,35,36]. The side effects of statins are widely spread because cholesterol biosynthesis is extremely complex and involves many reactions and components of the metabolic pathway (Figure 1) that can be important for the functioning of the body. The complete sequence of all cholesterol biosynthesis reactions was reproduced for the first time by the American biochemist R. Woodward, for which he was subsequently awarded the Nobel Prize in Chemistry [37]. Statins, by blocking cholesterol biosynthesis at the level of the enzyme HMG-CoA reductase, can cause a deficiency of certain substances that are necessary for the body. These include dolichol, vitamin D, bile acids, steroid hormones, lipoproteins, and ubiquinone (coenzyme Q), which is exceptionally important for all body cells, including cardiac myocytes. The following beneficial effects of coenzyme Q are most important for myocardial cells: energy (the most important component of the respiratory chain in cell mitochondria), antioxidant, and stabilization of cell membranes.

It is important to note that the mechanisms underlying pleiotropic and side effects have not been studied sufficiently, and in many aspects, there are a lot of discrepant data. This is largely due to the design of ongoing studies and the different research methods used. As for the methods of study, they are constantly being improved, while opening up new possibilities for researchers and changing the understanding of many pathological and physiological processes occurring in the body. The most illustrative examples and confirmation of this in the context of this paper are laboratory diagnostic methods, in particular, immunochemical assays for determining cardiac troponins (CTs) in blood serum, as well as methods of molecular genetic and ultramicroscopic studies. For example, the first methods for determining cardiac troponin-I (CT-I) and cardiac troponin-T (CT-T), developed by B. Cummins et al. in 1987 and H. Katus et al. in 1989, respectively [38,39], had extremely low sensitivity (high minimum detectable concentration 500–1000 ng/L and above) and could only detect large-focal myocardial infarctions late from the time of admission (12–24 h or more). Therefore, they were significantly inferior to the cardioenzyme creatine phosphokinase-MB isoform, which was then used as the gold standard. At the same time, CTs were considered strictly intracellular molecules, and their presence in blood serum was considered a pathological symptom indicating the death (irreversible damage) of cardiac myocytes. However, as the sensitivity of troponin immunoassays increased, the minimum detectable concentration decreased significantly to only a few ng/L or less in modern high- and ultra-sensitive immunoassays [40,41,42]. Thus, a newly developed assay provides the minimum detectable concentration of only 0.12 ng/L, which is more than a thousand times higher than the sensitivity of the very first troponin immunoassays and ten times more than high-sensitive first-generation assays [43]. This has allowed detecting serum CTs in all completely healthy patients. As a result, CTs are no longer considered strictly intracellular molecules, and provided that their concentration is less than the 99th percentile, they can be considered as normal metabolites of cardiac muscle tissue [40].

Moreover, high- and ultra-sensitive methods for determining CTs have raised the issue of the need to take into account the gender, age and circadian characteristics of CT concentration when using rapid algorithms for the diagnosis of acute myocardial infarction (AMI) [40,41,44,45,46,47]. Increased sensitivity has also contributed to the introduction of new data on the diagnostic capabilities of non-canonical to CVD biological fluids such as urine [48,49] and oral fluid [50,51,52,53], creating the foundation for further research on non-invasive diagnostic methods. 

## 3. Mechanisms of Statin-Induced Increase in CT Level

High-sensitive CTs, apparently, can increase in blood serum under the effect of a number of drugs and biologically active substances that stimulate the myocardium, such as cortisol, catecholamines and adrenomimetics, thyroid hormones. This is explained by the fact that circadian fluctuations in CT levels in healthy people coincide with circadian rhythms of endocrine glands producing these hormones [54,55,56,57,58,59]. Thus, cardiac myocytes began to be considered as cells that are extremely sensitive to the effect of various factors [60,61,62,63,64]. Some drugs, such as statins, are believed to have beneficial effects on the myocardium of patients, and therefore their effects on CT levels have not been widely studied [65,66,67]. Therefore, the data of S. Unlü et al. [68] reported a significant effect of statins on high-sensitive CT-T levels. The study included 56 patients, of which 26 were taking statins (experimental group) and 30 were not taking statins (control group). All study participants performed moderate physical exercise according to the established protocol. Blood samples were taken before the exercise and 4 h after. In those who took statins and exercised, the concentration of high-sensitive CT-T increased significantly after 4 h compared with initial indices (11.4 ± 15.2 ng/L versus 7.7 ± 12.6 ng/L, *p* = 0.004). In the control group of patients who did not take statins and only exercised, the level of high-sensitive CT-T was not increased after 4 h (7.74 ± 5.7 ng/L, versus 6.4 ± 3.5 ng/L versus *p* = 0.664) [68]. Thus, the concentration of high-sensitive CT-T in the experimental group was significantly higher than in the control group, which indicates a direct dependence on the use of statins by patients [68].

In addition, in some patients taking statins, the kinetics of high-sensitive CT-T elevation met the criteria for AMI diagnostic without ST segment elevation, approved by the European Society of Cardiology (ESC) [68,69,70].

The results obtained by S. Unlü et al. differ from the data of other researchers [71,72]. So, A. Trentini et al. studied the effect of statin drugs on different types of muscle fibers. Changes in the concentrations of slow and fast isoforms of skeletal troponins (ssTnI and fsTnI, respectively) and total creatine phosphokinase, as well as a marker of heart muscle damage—CT-I—were assessed. In patients taking statins, there was a specific increase in the concentration of fsTnI, but not ssTnI, which indicates that fast-twitch muscle fibers are most sensitive to the damaging effect of statins. In addition, patients who took statins often had a subclinical increase in fsTnI, but not in total creatine phosphokinase, which indicates a higher sensitivity and diagnostic significance of fsTnI at the early stages of the statin myopathy development. CT-I levels did not significantly differ in the experimental and control groups, which further confirms the selectivity of the statins damaging effect only on skeletal muscle fibers [71]. T Eijsvogels et al. studied the effect of statins on CT-I concentration in marathon runners 1 and 24 h after the finish. The use of statins did not significantly affect the amount of CT-I release (*p* = 0.47) or the number of runners whose CT-I level exceeded the diagnostic point to exclude AMI (57% versus 51%, *p* = 0.65). In addition, there was no significant association between statin dosages and cTnI release (r = 0.09, *p* = 0.65). Based on the results, the researchers concluded that marathon-induced increases in cTnI are not altered when using statins [72].

By analyzing the possible reasons for obtaining inconsistent results, it may be noted that there are differences in the design of the conducted studies, as well as methods for determining CTs. For example, the T Eijsvogels study participants were not examined for coronary heart disease, in contrast to the paper of S. Unlü et al. In addition, in two studies by A. Trentini and T Eijsvogels, CT-I concentration was determined with the usage of moderately sensitive methods, while S. Ünlü used a high-sensitive method to determine CT-T. The time of taking the biological material in patients after physical activity also differed. So, for example, taking the biological material too early (before 1 h) and too late (after 24 h), which was observed in the study by T. Eijsvogels, may not give an accurate idea of the CT level, since the moment of CT release is too early, and within a day, normalization of elevated CTs values in healthy patients is possible.

In addition, the sensitivity of conventional (moderately sensitive) methods for determining CTs may not be enough to detect subclinical damage to the myocardium by statins [71,72], while high-sensitive methods are able to register the fact of even the most insignificant damage [68,69,70]. Thus, statin therapy against the background of additional moderate physical activity not only has an adverse effect on the myocardium, but can also affect the accuracy of AMI diagnosis according to modern ESC diagnostic algorithms when using high-sensitive CT-T. On the other hand, it can be assumed that the study of S. Ünlü [68] cross-reacted anti-CT-T antibodies with skeletal troponin isoforms, which increased due to the myotoxic effect of statins. A similar cross-reaction was also recently found in a study where, in patients with various myopathies but no obvious symptoms of CVD, there was an increase in high-sensitive CT-T and high-sensitive CT-I in 68% and 4% of cases, respectively [73]. At the same time, it is very remarkable that non-specific (cross) reactions are most characteristic of high-sensitive methods for determining CT-T, which was just the same and was used in the study by S. Ünlü. This not only may explain the mechanism of the statin-induced increase in CT-T in this case, but also creates some concerns when using these high-sensitive immunoassays to diagnose AMI. 

Another very interesting case of increased CT levels after taking statins was described by English researchers P. Collinson and P. Kiely [74]. An elderly patient taking atorvastatin did not show any symptoms of acute CVD. However, high-sensitive CT-T levels were significantly elevated at 55 ng/L (99th percentile = 14 ng/L). Repeated (serial) measurements of high-sensitive CT-T also revealed elevated concentrations of high-sensitive CT-T (120 ng/L and 25 ng/L), but the concentrations of high-sensitive CT-I were within the normal range all this time (5 and 7 ng/L at the level of the 99th percentile = 30 ng/L). In the absence of any significant cardiac symptoms of an ischemic nature, the researchers concluded that there was a false-positive increase in the level of high-sensitive CT-T. In the process of further investigation by researchers, such causes as heterophilic antibodies and development of a macrotroponin system were excluded as possible false-positive factors [74]. Thus, the most likely mechanisms for increasing high-sensitive CT-T are either a direct cardiotoxic effect of atorvastatin on myocardial cells, or a false-positive reaction of anti-CT antibodies with skeletal troponin T molecules, which are released from skeletal muscle fibers due to their damage by atorvastatin. However, it is also worth noting that the latter mechanism is opposed by the fact that the authors of the study used modern certified troponin immunoassays, for which there were previously no similar cases of false-positive (cross) reaction of diagnostic antibodies with skeletal forms of troponins [74,75,76].

Damage to skeletal muscles under certain conditions (chronic renal failure [77], hereditary skeletal myopathies [77,78], and some types of glycogenosis (Pompe disease) [79]) may be accompanied by re-expression of CT molecules in skeletal muscle fibers and, accordingly, lead to an increase in the levels of these cardiospecific markers in serum by releasing the latter into the bloodstream. Hypothetically, it can be assumed that statin-induced skeletal muscle injury will also lead to extracardiac re-expression of CTs molecules. At the same time, it should be noted that this is a very controversial mechanism [80,81,82] and some researchers refute it [73,83].

In general, based on the analysis of the literature, the possible mechanisms for increasing CTs level can be summarized in Table 2.

## 4. Potential Mechanisms of Statins’ Toxic Effects on Myocardial Cells

Almost since their invention, statins have demonstrated a pronounced anti-atherogenic effect by lowering cholesterol levels and significantly reducing the risk of development and progression of atherosclerotic CVDs. In this connection, even the very idea of the possible negative effects of these drugs on the myocardium seemed extremely improbable. However, improved methods of laboratory diagnostics, histology and molecular biology have brought a lot of new data and views on this aspect. Thus, several experiments have reported the presence of potential cardiotoxic properties in statins [84,85,86,87,88]. J Godoy et al. studied ultrastructural changes in the myocardium of experimental animals under the action of statins (atorvastatin and pravastatin) and possible molecular mechanisms underlying the potential cardiotoxicity of statins. With long-term (7 months) oral administration of atorvastatin to rats, significant ultrastructural disorders of mitochondria were noted: swelling, change in size, displacement, and physical separation of mitochondria from each other, which in normal cardiomyocytes are all joined together using mitochondrial contacts, forming a chondriome like a three-dimensional network [84]. It is noteworthy that similar ultrastructural changes in mitochondria are also characteristic of skeletal muscle tissues during statin treatment [89], and in the heart muscle they are considered as the main symptom of metabolic changes that often precede the development of cardiac dysfunction [89,90,91,92]. Another statin drug, pravastatin, did not cause these negative changes in the myocardium of rats after long-term administration [84].

When studying the effect of statins on gene expression in cardiomyocytes, it turned out that atorvastatin, but not pravastatin, repressed the genes responsible for maintaining the integrity and proper functioning of mitochondria. In addition, researchers have found that atorvastatin, but not pravastatin, inhibits intracellular cardiac Akt/mTOR signaling [83], which, as was previously shown, regulates the physiological function of the heart and the survival of cardiac myocytes [85]. This raises the question of how long-term inhibition of mTOR upon administration of atorvastatin [84] will affect the integrity and survival of cardiac myocytes.

Atorvastatin also adversely affects the endoplasmic reticulum, causing endoplasmic reticulum stress in vitro and subsequent apoptosis of cardiomyocytes [93]. A study by S. Ghavami also reported that simvastatin induces endoplasmic reticulum stress and apoptosis in human atrial fibroblasts [93].

Y Zhu et al. [87] found that statin-induced depletion of membrane cholesterol in cardiomyocytes leads to a disruption in the architecture of the transverse (T) tubules, which are key structures for the conjugation of myocardial contraction processes. In addition to remodeling of cardiac myocytes T-tubules, by studying the myocardium morphology in an experimental model (isolated perfused myocardium according to Langendorff), the researchers found a violation of the integrity of intercalated discs (intercellular connections) between cardiac myocytes. These changes confirm previously found evidence that low serum cholesterol levels are associated with cardiac arrhythmias and poor prognosis in patients with chronic heart failure [87].

Statins can also affect the uptake of glucose by cardiac myocytes, while pravastatin and rosuvastatin, in contrast, have a less significant effect on this process [86]. Inhibition of insulin-induced glucose uptake by cardiac myocytes by atorvastatin can lead to a decrease in myocardial aerobic metabolism, since glucose is one of the main energy sources for the myocardium. 

Finally, as a final potential long-term mechanism of statin cardiotoxicity with respect to the myocardium, it is possible to consider the fact that statins increase the risk of developing diabetes mellitus [94,95], which in turn leads to micro- and macro-angiopathies, the development of CHD and gradual reduced delivery of blood and energy resources to cardiomyocytes.

Thus, statin-induced adverse myotoxic effects are characteristic of both skeletal and cardiac muscle tissue. Different statin drugs may have different effects at the molecular, genetic and cellular levels, inducing or suppressing certain intracellular signals that may enhance beneficial effects on the myocardium, and, on the contrary, weaken them and cause adverse changes. More experimental and clinical studies are needed for better understanding of the pleiotropic effects of statins and their clinical significance, which will subsequently be important in making decisions about the choice of a certain statin under certain conditions.

The main possible mechanisms of statins’ cardiotoxicity are summarized in Table 3.

Due to the inconsistency and insufficient knowledge of the existing data on the cardiotoxicity of statins, further studies using experimental simulation methods are needed to confirm or exclude the cardiotoxic effects of statins: the administration of various statin drugs with different dosages to laboratory animals, followed by a study of blood serum by high-sensitive methods for determining CTs, as well as assessing morphological changes in the myocardium at the ultrastructural level using electron microscopy.

## 5. Conclusions

Thus: taking into account the information available to date, it can be assumed that some statin drugs have cardiotoxic effects. This is confirmed by data on an increase in high-sensitive CT-T in those patients who take statins, as well as experimental papers on the study of cardiotoxic properties of statins at the ultrastructural and molecular levels. However, the available data are currently insufficient to conclusively confirm or refute the presence of cardiotoxic effects in statins. Further studies using high-sensitive laboratory and morphological methods will shed light on this aspect.

## Figures and Tables

**Table 1:** Criteria for inclusion and exclusion of articles for analysis.

*Inclusion Criteria*	*Exclusion Criteria*
Indexing: Scopus and/or WoS and/or PubMed/Medline databases	No indexing in Scopus and/or WoS and/or PubMed/Medline databases
Research type: original studies, clinical cases, experimental studies	Research type: reviews, comments, editorials
Object of research: people, animals, isolated heart (ex vivo), cardiomyocyte culture (in vitro)	Refusal to give informed consent
Characteristics of the object of study before the use of statins: stable condition of the disease (no recent exacerbations), not accompanied by a negative effect on cardiomyocytes	Characteristics of the object of study before the use of statins: acute cardiac and extra-cardiac pathologies (coronary heart disease, hypertension, pulmonary embolism, chronic obstructive pulmonary disease, etc. [15,16,17]), causing damage to cardiomyocytes
Research methods: laboratory diagnostics (determination of cardiomarkers, including cardiac troponins), morphological methods (light and/or electron microscopy), immunohistochemistry, molecular genetic methods	Research methods: without the use of laboratory and/or morphological and/or molecular genetic diagnostic methods

**Table 2:** Possible mechanisms for increasing CTs level after taking statins.

Mechanisms for Increasing	Comment
Cardiotoxic effects of statins	CTs increase due to the direct cardiotoxic effect of statins on myocardial cells
False-positive increase in CTs	CTs are increased due to the non-specific interaction of anti-CT antibodies with skeletal troponin molecules that are released due to statin-induced skeletal muscle damage (statin-induced myopathy)
Re-expression of CT molecules in skeletal muscles	CT molecules are re-expressed in damaged skeletal muscle due to the effect of statins and react with anti-CT antibodies when released into the bloodstream

**Table 3:** Main possible mechanisms of statins’ cardiotoxicity.

*Mechanism of Toxic Effect*	*Comment*	*Source*
Damage to the mitochondria of cardiac myocytes	Damage to mitochondria leads to inhibition of aerobic glycolysis in cardiac myocytes and, as a result, to a decrease in the production of ATP energy, which is necessary for proper functioning and maintaining of the structural integrity of myocardial cells	[84,89]
Stress of the endoplasmic reticulum	Violation of the endoplasmic reticulum functioning is accompanied by a violation of cardiac myocytes calcium homeostasis, the maintenance of which is associated with the regulation of contraction-relaxation of the myocardium	[84,93]
Ubiquinone deficiency	Ubiquinone, being an essential component of the mitochondrial respiratory chain, is exceptionally important in terms of ATP molecules generation in cardiac myocytes	[31,32]
Induction of cardiac myocytes apoptosis	Statin-induced apoptosis of cardiac myocytes can develop due to a number of factors: (1) energy deficiency as a manifestation of mitochondrial damage and a decrease in ubiquinone formation; (2) imbalance of calcium homeostasis as a manifestation of endoplasmic reticulum stress; (3) inhibition of the anti-apoptotic Akt/mTOR pathway in myocardial cells	[84,85,89,93]
Deformation of intercalated discs between cardiac myocytes	Excessive cholesterol reduction due to statin use can lead to violation of the structure and functioning of those cellular structures which contain this lipid in large quantities, in particular, the plasma membrane, T-tubules, intercalated discs of cardiac myocytes	[87]
Inhibition of glucose intake into myocardial cells	Since glucose is the most important energy substrate for myocardial cells, limitation of its intake may be accompanied by a decrease in the formation of ATP energy in cardiac myocytes	[86]
Diabetogenic (hyperglycemic) effect	This effect of statins may, in the long term, contribute to the development of diabetic angiopathy, including those involving the coronary arteries, due to which the delivery of oxygen and metabolic products through them will be reduced, which can lead to subclinical damage and the release of the cytoplasmic CT fraction	[94,95]
